# FOXO3 Transcription Factor Regulates IL-10 Expression in Mycobacteria-Infected Macrophages, Tuning Their Polarization and the Subsequent Adaptive Immune Response

**DOI:** 10.3389/fimmu.2019.02922

**Published:** 2019-12-12

**Authors:** Rania Bouzeyen, Meriam Haoues, Mohamed-Ridha Barbouche, Ramandeep Singh, Makram Essafi

**Affiliations:** ^1^Laboratory of Transmission, Control and Immunobiology of Infections (LTCII), Laboratoire de Recherche 11 (LR11), Institut Pasteur de Tunis (IPT), Tunis, Tunisia; ^2^Université Tunis El Manar, Tunis, Tunisia; ^3^Tuberculosis Research Laboratory, Translational Health Science and Technology Institute, NCR Biotech Science Cluster, Faridabad, India

**Keywords:** tuberculosis, macrophages, FOXO3, IL-10, M1/Th1

## Abstract

Alveolar Macrophages play a key role in the development of a robust adaptive immune response against the agent of Tuberculosis (TB), *Mycobacterium tuberculosis* (*M.tb*). However, macrophage response is often hampered by the production of IL-10, a potent suppressor of the host immune response. The secretion of IL-10 correlates with TB pathogenesis and persistence in host tissues. Concordantly, inhibition of IL-10 signaling, during BCG vaccination, confers higher protection against *M.tb* through a sustained Th1 and Th17 responses. Therefore, uncovering host effectors, underlying mycobacteria-induced expression of IL-10, may be beneficial toward the development of IL-10-blocking tools to be used either as adjuvants in preventive vaccination or as adjunct during standard treatment of TB. Here, we investigated the role of FOXO3 transcription factor in mycobacteria-induced secretion of IL-10. We observed that PI3K/Akt/FOXO3 axis regulates IL-10 expression in human macrophages. Knocking down of FOXO3 expression resulted in an increase of IL-10 production in BCG-infected macrophages. The gene reporter assay further confirmed the transcriptional regulation of IL-10 by FOXO3. *In silico* analysis identified four Forkhead binding motifs on the human IL-10 promoter, from which the typical FOXO3 one at position −203 was the major target as assessed by mutagenesis and CHIP binding assays. Further, we also observed a decrease in gene expression levels of the M1 typical markers (i.e., CD80 and CD86) in SiFOXO3-transfected macrophages while activation of FOXO3 led to the increase in the expression of CD86, MHCI, and MHCII. Finally, co-culture of human lymphocytes with siFOXO3-transfected macrophages, loaded with mycobacterial antigens, showed decreased expression of Th1/Th17 specific markers and a simultaneous increase in expression of IL-4 and IL-10. Taken together, we report for the first time that FOXO3 modulates IL-10 secretion in mycobacteria-infected macrophage, driving their polarization and the subsequent adaptive immune response. This work proposes FOXO3 as a potential target for the development of host-directed strategies for better treatment or prevention of TB.

## Introduction

Tuberculosis (TB) remains one of the top causes of death with an estimated 1.4 million deaths and 10.4 million new cases worldwide (1). The global TB burden has increased due to emergence of drug-resistant *Mycobacterium tuberculosis* (*M.tb*) strains and the limited protection conferred by *M. bovis* BCG against pulmonary TB (2). Targeting the host effectors, involved in TB immune response, has been proposed as a viable adjunct therapy for elimination of both drug-sensitive and drug-resistant TB (3) or to enhance the BCG protective efficacy (4). Therefore, there is a critical need to uncover the TB host responsive clues that might lead to the development of novel host-directed approaches for better treatment or prevention of TB.

Macrophages are major innate immune cells, which play key roles in TB infection as an intracellular niche and serve as a first line defense against *M.tb* infection (5). Phagocytosis of mycobacteria initiates a series of innate and adaptive immune responses to contain the infection (6). Importantly, macrophage-derived cytokines, such as TNF-α, IL-12, and IL-1 family members as well as chemokines and antimicrobial peptides (AMPs) are critical for host anti-mycobacterial defense and shaping the disease progression (7). However, macrophage response is often hampered by the production of IL-10, a potent suppressor of the host immune response, which was reported to be correlated with TB pathogenesis and persistent of infection in humans (8, 9). Several studies have shown that IL-10 plays an important role in shaping the initial immune response and its expression level determines the fate of mycobacterial infections. Indeed, induction of IL-10 by *M.tb*-infected macrophages and dendritic cells (DC) represents a powerful mechanism of immune evasion (10). It has been reported that *M.tb*-induced IL-10 secretion interferes with the phago-lysosome fusion, macrophage activation, and resulting in persistence of the invading bacteria (11). It has been shown that IL-10 affects antigen procession and T cells priming by downregulating the expression of the major histocompatibility complex class II, CD80, CD86, and CD11c in BCG-infected macrophages and DC (12, 13). IL-10 also inhibits the production of pro-inflammatory cytokines, and the release of reactive nitrogen and oxygen intermediates by macrophages, thereby leading to a weakened Th1/Th17 and an enhanced Th2 response (7, 14, 15). Likewise, *M.tb*-induced IL-10 promotes M2 macrophages polarization, which displays dampened anti-mycobacterial response (16).

On the other hand, IL-10 suppression promotes mycobacterial clearance by the host during the early stages of *M.tb* infection (15). IL-10 deficient mice display an enhanced Th1 immune response to aerosol challenge with *M.tb* and lower bacterial burden as compared to the wild type mice (17). In concordance, the susceptible mice strains such as the CBA/J express higher levels of IL-10 in chronic infection, while blocking of IL-10 signaling resulted in lower bacterial load and increased mice survival (18). Conversely, the artificial enhancement of IL-10 expression in a resistant mouse strain increased their susceptibility to *M.tb* infection (19). These studies suggest that IL-10 production is correlated with susceptibility to *M.tb* and suppression of the protective immune response in mice. In concordance, active TB patients present elevated levels of IL-10 in the lungs serum, sputum and bronchoalveolar lavage fluid (BAL) (20). Moreover, it has been reported that macrophages from TB patients produce higher level of IL-10 than the ones from healthy subjects (8, 21, 22). Likewise, patients with MDR-TB have an altered balance between Th1/Th2 response with decreased Th1 associated cytokines and increased IL-10 secretion (22). High level of IL-10, at the end of treatment in pulmonary TB patients, was also associated with TB recurrence (7), indicating that IL-10 plays an essential role in TB pathogenesis and disease progression. Inhibition of IL-10 signaling, during BCG vaccination, enhances antigen-specific IFN-γ and IL-17 responses and results in better protection against *M.tb* challenge (23). These findings demonstrate that modulation of IL-10 at the time of vaccination could be crucial for generating long-term protective efficacy against *M.tb* (24). Therefore, identifying host effectors that regulate mycobacteria induced IL-10 secretion might be useful for the development of host-directed therapy approaches against *M.tb*.

Previous studies have identified two important signaling cascades that control IL-10 expression in macrophages, the PI3K/Akt/GSK3b and JAK/STAT3 pathways (14). Pharmaceutical inhibition of PI3K/AKT in macrophages impairs IL-10-induced gene expression and IL-10-mediated suppression of LPS-induced pro-inflammatory gene (25). Interestingly, It has been demonstrated that BCG induces IL-10 production through PI3K/AKT signaling while prevention of IL-10 secretion and enhancement of IL-12 production is associated with a decreased Akt phosphorylation (26). In another study, it has been reported that *M.tb* evades macrophage microbicidal activity by activating the Akt/mTOR/p70S6K pathway, while inhibition of Akt phosphorylation results in reduced *M.tb* intracellular survival (27).

Forkhead box-O3 (FOXO3) protein is a transcription factor that regulates the expression of multiple genes involved in pathways such as cell-death, cell proliferation, oxidative stress resistance, inflammation, and innate immune homeostasis (28). We have previously reported that BCG-mediated apoptosis of human macrophages relies on FOXO3 activation, which is negatively regulated by the survival pathway PI3K/Akt (29). We demonstrated that BCG infection of macrophages resulted in Akt dephosphorylation and its target FOXO3. The dephosphorylated FOXO3 is subsequently translocated to the nucleus and induced the expression of the pro-apoptotic effectors NOXA and PUMA (29). In concordance, in comparison to healthy controls, low expression of FOXO3 in active TB patients is associated with a defect in monocytes apoptosis (30). The fact that high level of IL-10 expression is also a hallmark of TB pathogenesis (8, 9) suggests FOXO3 as a potential repressor of IL-10 secretion.

Here, we studied whether FOXO3 regulates IL-10 secretion in mycobacteria-infected macrophages and how this regulation would influence the adaptive immune response against mycobacterial infection. We show that FOXO3 inhibits IL-10 secretion in BCG-infected macrophage through the direct binding to and repression of IL-10 promoter, pushing the balance of the immune response toward an M1/Th1 phenotype. These observations suggest FOXO3 as a potential target to develop alternative host-directed approaches for better treatment and prevention of TB.

## Materials and Methods

### Cell Culture

The THP-1 cell line (American Type Culture Collection, ATCC TIB-202) was maintained and cultured as described previously (29). Briefly cells were maintained in RPMI 1640 (Thermo Fisher, USA) supplemented with 10% fetal bovine serum (FBS) (Thermo Fisher, USA), 12 mM HEPES, 0.1 mM MEM non-essential amino acids, 1 mM Sodium Pyruvate, and 100 nM penicillin/streptomycin (Life Technologies). THP-1 monocytes were differentiated to macrophages (TDMs) with 20 ng/mL phorbol 12-myristate 13-acetate (PMA, Sigma-Aldrich, USA) for 48 h. RAW264.7 (ATCC TIB-71) and J774A.1 (ATCC TIB-67) murine macrophage cell lines were also maintained in RPMI medium. Peripheral blood mononuclear cells (PBMCs) were isolated from three healthy volunteers as approved by the institutional ethics committee of Institut Pasteur de Tunis. These donors were negative for any recent infection and had no history of TB. Briefly, cells were differentiated into macrophages derived monocytes (MDMs) and cultured as previously described (29). Macrophages were infected with a single cells suspension of BCG Pasteur strain, at a multiplicity of infection (MOI) of 1:10 for 3 h. The extracellular bacteria was removed by washing twice with 1× PBS and overlaid with medium containing inhibitors for different time points. The drugs were used at the following concentration: Wortmannin (100 nM) (Sigma-Aldrich, USA) or MK-2206 (5 μM) (Selleckchem, USA). It is worth to note that we avoided adding the inhibitors before BCG infection because PI3K/Akt pathway has been shown to control phagocytosis. Supernatants were collected 24 h post-infection and frozen at −20 until use. The cells were harvested 24 h post-infection for western blot or Real-time quantitative RT-PCR (RT-qPCR) analysis.

### Cell Toxicity Assay

Cytotoxicity of the inhibitors used in this study was assessed using WST-1 kit (Sigma Aldrich, USA) as per the manufacturer's instructions. Briefly, cells (2.10^4^/well in a 96-well plate) were treated with different concentrations of the inhibitors, or with equivalent volume of vehicle control. Twenty-four hours post treatment, media was removed, substrate was added and plate was further incubated at 37°C for 30 min. The optical density values were determined by a microplate reader at 450 nm and the values were normalized with those of untreated cells. All experiments were carried out in triplicates.

### RNA Interference Experiments

TDMs or MDMs were transfected, using the HiPerfect Transfection reagent (Qiagen), with either 100 nM control non-silencing siRNA (SiCT), or with 50 nM FOXO3 siRNAs (siGENOME SMARTpool, Thermo Fisher Scientific) according to the manufacturer's instructions. After 48 h of transfection, cells were infected with *M. bovis* BCG at an MOI of 1:10. After 24 h of infection, cells were harvested and the knockdown of FOXO3 was verified by both Western blot and RT-qPCR analysis.

### Western Blotting

For immunoblot analysis, macrophages were lysed in 1× laemmli buffer and the protein concentration was determined using Bicinchoninic Acid Protein Assay (BCA, Sigma-Aldrich). Equal amounts of protein was separated by electrophoresis on 10% SDS-PAGE gel, transferred to PVDF membrane and subsequently probed with the respective antibodies as per manufacturer recommendations. In the present study, the following primary antibodies were purchased either from Sigma Aldrich, Merck [FKHRL1-D12 (p-FOXO3^Thr32^), FKHRL-1 (FOXO3), β-actin] or from Cell Signaling Technology, USA (Akt and p-Akt^Ser473^). Western blots were quantified by densitometric analysis using Image J software.

### Real-Time Quantitative RT-PCR

Total RNAs from treated cells were isolated using RNeasy Micro kit (Qiagen), and the cDNAs were synthesized by reverse transcriptase (RT) using the high capacity cDNA archive random priming kit (Applied Biosystems) according to the manufacturer's recommended protocol. Real-time quantitative PCR was performed using the platinum® Syber Green qPCR Supermix-UDGw/ROX (life technologies). The relative expression of the target genes was calculated using the target threshold cycle value (Ct) and the 2^−ΔΔ*Ct*^ method with β-actin and GAPDH as the internal loading controls. The primer sequences used for real time PCR are listed in Tables S1, S2.

### ELISA Cytokine Assays

For cytokine assays, supernatants were collected at different time points post BCG infection and used to measure IL-10, TNF-α, IFN-γ, and IL-6 concentrations using ELISA assay kits as per manufacturer recommendations (BD Biosciences).

### *In silico* Analysis and Gene Reporter Assay

To identify putative FOXO3 binding sites, the human IL-10 promoter region spanning from −560 to +1 was analyzed *in silico* using the MatInspector analysis tool of the Genomatix software (https://www.genomatix.de/online_help/help_matinspector/matinspector_help.html). For reporter assays, the construct pGL3-IL-10, containing the −588 IL-10 promoter sequence upstream of the luciferase transcriptional unit of the pGL3 vector was used. This construct was a gift from John W. Steinke, Asthma and Allergic Diseases Center, Charlottesville VA, USA. Transient transfections into RAW 264.7 macrophages were performed using the Amaxa SF Cell Line 4D-nucleofector Kit (Lonza Köln, Germany) as per manufacturer recommendations. Briefly, 1 × 10^6^ macrophages were washed and resuspended in 20 μl of transfection buffer and mixed with 1 μg of pEGFP vector, encoding for GFP or pEGFP-FOXO3TM construct, encoding for GFP-FOXOTM (constitutive active form of FOXO3 where the three Akt binding sites were mutated) along with the reporter pGL3-IL-10 construct or the pGL3 empty vector). Cells were then transfected using program Raw264.7 on Lonza 4D-nucleofector device. Cells were rapidly transferred to 37°C preheated medium and incubated for 24 h at 37°C, 5% CO_2_. Twenty-four hours post-transfection, macrophages were either mock-treated or infected with BCG at an MOI of 1:10. After 6 h, macrophages were assessed for viability using Annexin-V staining and the remaining cells were harvested, lysed and luciferase activity was measured using Thermo Scientific Varioscan Flash Plate reader as per manufacturer's recommendations (Promega, Madison, WI, USA). The promoter activities are shown as relative light unit (RLU) and normalized to 100% GFP positive and viable cells (Figure S2) using the following equation:

(1) $$\begin{array}{r} \text{Promoter activity}=\frac{\text{RLU}\;x\;100}{\%\;\text{GFP}\;\text{positive}\;\text{and}\;\text{viable}\;\text{cells}} \end{array}$$

### Site Directed Mutagenesis of the IL-10 Promoter

The four FOXO3 binding sites in −588 IL-10 promoter construct were mutated using Quick-change XL site directed mutagenesis kit (Agilent technology) as per manufacturer's recommendations. The list of primers used to disrupt FOXO3 motifs mutagenesis is shown in Table S3. The mutant 1, 2, 3, and 4 were constructed by deleting CAC (position −17), GCT (position −203), AAT (position −303), and TTA (position −526), respectively. The effect of each mutation on transcriptional activity of IL-10 promoter was assessed by gene reporter assay as indicated above.

### Chromatin Immunoprecipitation (CHIP)

Next, we performed CHIP assay in TDMs using the Chromatin Immunoprecipitation (CHIP) Kit (Millipore) according to the manufacturer's instructions. Briefly, macrophages were infected with BCG at an MOI of 1:10 for 2 h, washed thrice with 1× PBS and overlaid with medium containing 100 nM wortmannin for 2 h to enhance FOXO3 translocation to the nucleus. TDMs were subsequently crosslinked with 1% formaldehyde at 37°C for 10 min and quenched with 0.2 M glycine for 10 min. The crosslinked cells were washed twice and lysed in cell lysis buffer. Subsequently, chromatin was sheared by sonication at 60% power for 10 cycles, 10 s ON/1 min off, to obtain 100–1,000 bp chromatin fragments. For immunoprecipitation, the sheared chromatin was diluted 10 times, precleared with Protein A/G agarose salmon sperm DNA (Millipore) for 30 min at 4°C, then incubated with rabbit anti-FKHRL1 (FOXO3) sc-11351X, Santa cruz Biotechnology) or an equal amount of rabbit anti-IgG (sc-2027, Santa cruz Biotechnology) at 4°C overnight. The Antibody/DNA complexes were co-immunoprecipitated with protein A/G agarose at 4°C for 1 h. The immunoprecipitated DNA was eluted and crosslinking was reversed by incubation at 65°C for 4 h in 5M NaCl. The mix was later incubated with 10 mg/mL of proteinase K at 45°C for 1 h and the DNA from starting (1% input) and immunoprecipitated samples were purified by phenol-chloroform extraction and ethanol precipitation. The resulting pellets were suspended in H_2_O, quantified and subjected to PCR using primers (F1 5′- CCTCTGCGCACA GAACAGCTG-3′ and R1 5′- CTAACCTCTCTAATAAACTTAG-3′) flanking the FOXO3 binding motif at −203 position.

### Effect of FOXO3 on Macrophage Polarization and Adaptive Immune Response

In order to evaluate the effect of FOXO3 on macrophage polarization and adaptive immune response, human monocytes were transfected either with control or with FOXO3 specific siRNA for 48 h before the addition of autologous lymphocytes (purified after depletion of the monocytes from total PBMC) along with the mycobacterial purified protein derivative (PPD). After 96 h, secreted cytokines were quantified in supernatants by ELISA. Further, total RNA was extracted from whole cells and the gene expression profiles of M1/M2/Th1/Th2/Th17 markers were measured by qPCR. In addition, the effect of FOXO3 activation (through Akt inhibition) on macrophage polarization was also assessed in BMDMs. BMDM's were isolated from 6 to 8 weeks old female BALB/c mice as per standard protocols and resuspended in DMEM supplemented with 20% FBS, 20% L929 supernatant medium, 1% L-glutamine, 1% Pen/Strep, 1× MEM, and 1% sodium pyruvate. The BMDM's were incubated for 6 days at 37°C and 5% CO_2_. For experimental setups, BMDMs were seeded in a 24 well plate at a density of 5 × 10^5^ cells per well, infected with BCG at an MOI of 1:10 and subsequently overlaid with medium without or with 5 μM of MK-2206. Twenty-four hours post infection, BMDMs were harvested, washed and incubated for 10 min with anti-CD16/CD32 to block non-specific antibody binding. Subsequently, cells were stained with anti-CD11b (BV510), anti-CD86 (PEcy7), anti-MHC class II (PB515), anti-MHC class I (BV421) for 30 min at 4°C as per manufacturer's recommendations (BD Biosciences). The antibody-stained cells were washed twice with ice cold 1× PBS, suspended in FACS staining buffer and data was collected using flow cytometry (FACS CantoII, BD Biosciences). The acquired data was analyzed with Flow Jo software.

### Statistical Analysis

Statistical analyses and generation of graphs were performed with GraphPad Prism version 7. Data are expressed as the mean ± standard error of the mean (SEM). Comparison between groups was performed by a paired *t*-test. Differences with a *p* < 0.05 were considered statistically significant. Data generated using blood samples from the three human volunteers were further analyzed by the one-tailed version of the Mann Whitney test, which is more suited for small-sized samples (31, 32).

## Results

### PI3K/Akt/FOXO3 Axis Regulates IL-10 Expression in BCG-Infected Macrophages

In macrophages, IL-10 release has been shown to be dependent on PI3k/Akt activation (14). Since FOXO3 is a major target that is directly phosphorylated/inhibited by Akt (33), we sought to investigate whether the pharmacologically inhibition of PI3K/Akt signaling pathway, have an impact on FOXO3 activity and BCG-induced secretion of IL-10. To determine this, we used Wortmannin (Wort), one specific PI3K inhibitor, and MK-2206 (MK), a selective non-ATP-competitive allosteric Akt inhibitor (34). We first assessed the cytotoxicity of both inhibitors on human and murine macrophages using the WST1 test. No significant cytotoxicity was associated with different concentrations of MK-2206 in both TDMs and J774A.1 cells as >90% of both cell lines survived at concentrations lesser or equal to 5 μM (Figure S1). Wort treatment produced significant toxicity only at concentrations above 100 nM in TDMs cells, while, no cytotoxicity was observed in J774A.1 cells following exposure to increasing concentrations of Wort (Figure S1). Hence, for the subsequent experiments we used MK at a concentration of 5 μM and Wort at 100 nM.

**Figure 1:**
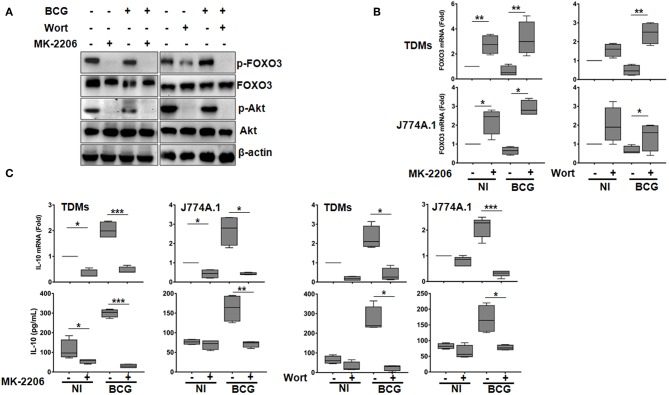
The PI3K/Akt axis regulates BCG-induced IL-10 secretion in macrophages. Human TDMs and mice J774A.1 macrophages were mock-treated or infected with BCG at an MOI = 10 for 3 h, washed then treated or not with the PI3K inhibitor, Wortmannin (Wort, 100 nM), or with the Akt inhibitor, MK-2206 (5 μM) for 24 h. **(A)** Inactivation of PI3k/Akt axis was determined, 24 h post infection/treatment, in whole cell lysate of TDMs by western blotting using the indicated anti-phospho specific antibodies. Total Akt, FOXO3, and β-actin were used as loading controls. Shown are representative images of three independent experiments with similar results. **(B)** FOXO3 mRNA expression were quantified by qRT-PCR in both TDMs and J774A.1 macrophages. Levels of mRNA were normalized to GAPDH and fold induction was calculated relative to uninfected and untreated cells. **(C)** Assessment of IL-10 transcription and secretion levels, after the indicated treatments, were quantified by qRT-PCR and sandwich ELISA, respectively. For all panels, results are represented in box and whiskers plot format min to max and are representative of three independent experiments, each one carried out in triplicate. Asterisks indicate statistical significance (*p < 0.05; **p < 0.01; ***p < 0.001).

We found that treatment of TDMs macrophages with both Wort and MK resulted in Akt dephosphorylation in both BCG-infected and non-infected cells. In concordance with our hypothesis, we also observed that treatment with these drugs resulted in FOXO3 dephosphorylation (Figure 1A), indicating its activation. Interestingly, MK and Wort treatment also induced a significant up-regulation of FOXO3 gene expression level in BCG-infected and non-infected TDMs and J774A.1 macrophages, indicating that PI3K/Akt pathway also inhibits FOXO3 activity by abrogating its transcription (Figure 1B).

Concomitantly, RT-qPCR showed that infection of macrophages with BCG increased levels of IL-10 mRNA, which were significantly abrogated upon MK and Wort treatment in both mice and human macrophages (Figure 1C). Consistent with RT-qPCR data, ELISA quantification revealed a significant inhibition (about 90%), by Wort (*p* < 0.05) and MK (*p* < 0.001) inhibitors of IL-10 secretion in BCG-infected TDMs. Similar results were obtained in BCG-infected mouse macrophages J774A.1 (Figure 1C).

These observations clearly indicates that PI3K/Akt pathway positively regulates IL-10 expression in BCG-infected macrophages and that such regulation might rely on FOXO3 inhibition. It is worth to note that we have also verified the effect of the PI3K/Akt/FOXO3 axis on the expression of TNF-α, a cytokine that plays a crucial role in TB immune response, and found that neither the used inhibitors nor the FOXO3 silencing had an effect on BCG-induced secretion of TNF-α (Figure S3).

**Figure 2:**
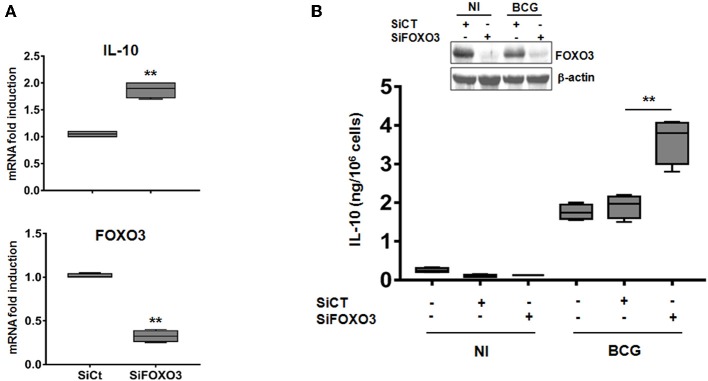
Knockdown of FOXO3 augments IL-10 expression in BCG-infected human primary macrophages. MDMs were transfected with FOXO3 specific siRNA (SiFOXO3) or with scramble control siRNA (siCT) for 48 h and then infected with BCG for 24 h. **(A)** Fold induction levels of FOXO3 and IL-10 mRNAs were assessed by RT-qPCR in BCG-infected cells. **(B)** IL-10 secretion by the indicated treated cells was assessed in the culture supernatants by ELISA. Knocking down of FOXO3 expression was further verified by immunoblotting. Data are shown as box and whiskers (min to max values) and are representative of three independent experiments, each one carried out in triplicate (*n* = 3, **p < 0.01).

To further investigate the role of FOXO3 on IL-10 expression in mycobacteria-infected macrophages, we transfected human MDMs with a pool of small interfering RNA (siRNA) targeting the endogenous expression of FOXO3. The knockdown efficiency of FOXO3 was confirmed by RT-qPCR and western blotting (Figure 2). We found that silencing of FOXO3 significantly enhanced the BCG-induced transcription of IL-10, compared to the scrambled Si control (SiCT)-transfected cells (Figure 2A), suggesting FOXO3 as an inhibitor of IL-10 transcription. In agreement, we observed an increase of BCG-induced secretion of IL-10 in SiFOXO3-transfected macrophages (Figure 2B). Taken together, these data suggest that FOXO3 negatively regulates IL-10 expression in BCG-infected macrophages.

### FOXO3 Binds and Inhibits the Transcription From IL-10 Promoter

**Figure 3:**
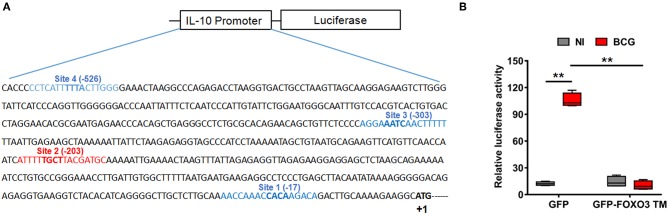
FOXO3 inhibits IL-10 promoter activity in BCG-infected macrophages. **(A)** Schematic representation of the four putative Forkhead DNA-binding elements (FKHR-DBEs) in the proximal region of the human IL-10 promoter (-588), identified by Genomatix algorithm. The sequence in red is a typical FOXO3 binding motif. **(B)** RAW 264.7 cells were co-transfected with pGL3 plasmid containing the −588 bp IL-10 promoter region fragments plus either the GFP or GFP-FOXO3-TM-encoding vectors. Twenty-four hours later, cells were infected or not with BCG (MOI ~10) for 6 h before measuring the Luciferase activity in GFP positive alive cells. The data correspond to the mean ± SEM and correspond to a one representative of two independent experiments, each one conducted in triplicates (*n* = 2, **p < 0.01).

**Figure 4:**
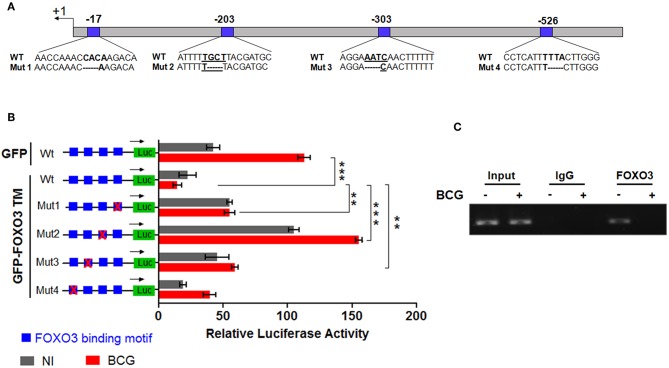
FOXO3 binds to IL-10 promoter, inhibiting its transcriptional activity in BCG-infected cells. In order to determine the contribution of each Forkhead DNA-binding element identified *in silico*, four mutants of IL-10 promoter were generated as indicated in **(A)**. **(B)** representation of gene reporter assay performed on the wild type IL-10 promoter and the four mutants (Mut1, Mut2, Mut3, Mut4). RAW 264.7 macrophages were transfected with the indicated constructs. Twenty-four hours later, cells were infected or not with BCG and luciferase activity was measured 6 h later in GFP positive alive cells. The data correspond to the mean ± SEM of three different experiments conducted in triplicates. Asterisks indicate statistical significance (*n* = 3, ** *p* < 0.01, *** *p* < 001). **(C)** TDMs were infected with BCG for 3 h and treated with Wort for additional 2 h. Next, CHIP assay was conducted to verify FOXO3 recruitment to the site −203 of the IL-10 promoter with either anti-FOXO3 Ab (lane 1 and 2) or anti-IgG isotype Ab (lane 3 and 4). Total input of BCG infected and non-infected cells are shown in lanes 5 and 6.

To evaluate whether IL-10 is subjected to direct transcriptional control by FOXO3, we performed an *in silico* analysis of the human IL-10 promoter region using the MatInspector program of the Genomatix portal (https://www.genomatix.de/online_help/help_matinspector/matinspector_help.html). Scanning for Forkhead DNA-binding elements (FKHR-DBEs) on the Genomatix-proposed IL-10 promoter sequence revealed three Forkhead putative motifs at positions −17, −303, and −526 and one typical FOXO3-response elements at position −203 (Figure 3A and Table S4). Next, we performed a gene reporter assay to understand FOXO3-mediated regulation of IL-10 promoter activity as described in methods. As shown in Figure 3B, we observed that infection of macrophages with BCG increased the IL-10 promoter activity by ~8.0 folds in comparison with non-infected cells (*p* = 0.0010). However, overexpression of FOXO3 almost abolished the BCG-induced IL-10 promoter activity in macrophages (Figure 3B, 90% inhibition, *p* = 0.025). In order to better explore the functionality of FKHR-DBEs and determine which motifs are involved in FOXO3 mediated inhibition of IL-10 promoter activity, we mutated the four putative sites and performed the luciferase assay (Figure 4A). We found that disruption of the FKHR binding elements at site 1 (position−17) and site 3 (position −303) partially elevated the FOXO3-mediated inhibition of IL-10 promoter activity, while the mutation of site 4 (-526) did not affect the inhibition of BCG induced IL-10 promoter activity by FOXO3. However, mutation of the typical FOXO3 motif at Site 2 (position −203) showed almost a total unleash of the FOXO3-mediated inhibition of IL-10 promoter (Figure 4B). These results suggest that FOXO3-mediated down regulation of IL-10 in BCG-infected macrophages mainly occurs through site 2 on which FOXO3 might bind and block the transcription.

To confirm our observation, we performed CHIP followed by a PCR using two primers flanking the −203 position, as indicated in materials and methods section. The result revealed a strong interaction between FOXO3 and the amplified region containing the motifs at −203 in non-infected cells, while BCG infection displaced FOXO3, releasing IL-10 transcription (Figure 4C). This data correlates with the observed higher basal activity of IL-10 promoter that bears a mutated site 2 (-203) (Figure 4B). Taken together, we demonstrate that FOXO3 transcription factor binds to IL-10 promoter at position −203 and negatively regulates its expression.

### FOXO3 Tunes Phenotypic Maturation of Mycobacteria-Infected Macrophages

**Figure 5:**
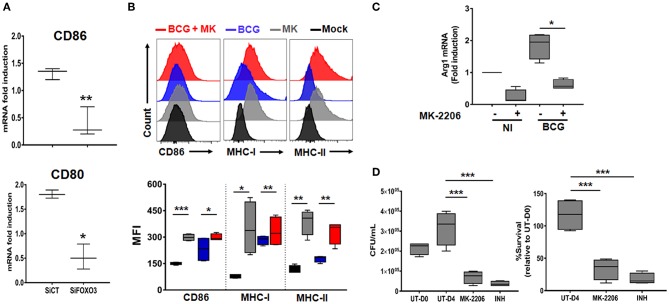
FOXO3 tunes phenotypic maturation of mycobacteria-infected macrophages. **(A)** Human monocytes were transfected either with SiCT or SiFOXO3 for 48 h followed by PPD stimulation and incubation with the autologous lymphocytes for 4 days. Whole cells were then collected and tested for the expression levels of CD80 and CD86 by qRT-PCR. Data are presented as box and whiskers graphs (min to max) from three independent experiments (i.e., MDMs from 3 different donors), each one carried out in triplicate (*n* = 3, **p < 0.001, ***p < 0.0001). **(B)** Non-infected or BCG-infected BMDMs were treated or not with MK-2206 (5 μM) for 24 h. The expression levels of the M1 phenotype surface markers CD86, MHC-MHC-II, and MHC-I, in CD11b^**+**^ cells, were determined by FACS analysis using specific antibodies. FACS analysis was performed on two independent experiments and each sample was run in triplicates (*n* = 2, *p < 0.05, **p < 0.001, ***p < 0.0001). **(C)** J774A.1 were mock or infected with BCG at an MOI = 10 for 3 h, washed then treated or not with the Akt inhibitor MK (5 μM) for 24 h. The transcript levels of M2 marker, Arg1, was assessed by RT-PCR. Data are presented as box and whiskers (min to max) and are from two separate experiments, each one carried out in triplicate (*n* = 2, *p < 0.05). **(D)** TDMs were infected with BCG at an MOI = 10 for 3 h. Following incubation, cells were treated with Amikacin (200 μg/ml) for 1 h to remove extracellular bacteria. The infected cells were then washed and treated with MK (5 μM) for 4 days. The antibiotic Isonicotinylhydrazide (INH) was used as positive control. Cells were lysed with sterile PBS containing 0.1% triton X100 and 10-fold serial dilutions of lysates were plated on MB7H11 for CFU Enumeration. The Intracellular colony forming units were determined at the indicated time points. UT-D0, untreated at Day 0; UT-D4, untreated at Day 4. Data are shown as box and whiskers (min to max values) and representative of two separate experiments, each one carried out in quadruplets. Asterisks indicate statistical significance (*n* = 2, ***p < 0.0001).

During the early phase of *M.tb* infection, the host generates the pro-inflammatory M1 type macrophages, which are associated with protection against the disease (35). As the infection progresses, the pathogen induces the re-polarization of macrophages toward the immunomodulatory M2 phenotype (36). Such transition is driven by high levels of Th2 cytokines such as IL-10 and correlates with disease severity (37). In order to assess the impact of FOXO3-mediated inhibition of IL-10 expression on macrophage phenotype, we quantified the transcription levels of the two typical M1 surface markers CD80 and CD86 in PPD-treated and non-treated human macrophages, as detailed above (Figure S4). We found a significant decrease of the PPD-induced expression of the two studied markers after FOXO3 silencing in human macrophages, compared to the Sicontrol-transfected ones (Figure 5A). Conversely, pharmacological activation of FOXO3 by MK, in BCG-infected and non-infected BMDMs, resulted in the up-regulation of CD86 expression level along with the ones of MHC class II and class I, two presenting molecules and markers of M1 macrophages (Figure 5B). MK treatment has also significantly decreased the expression levels of the typical marker of M2 macrophages, Arg1 (Figure 5C). These data suggest that FOXO3-mediated inhibition of IL-10 enhances the antigen-presenting activity of macrophages and favors their polarization toward the pro-inflammatory M1 phenotype during TB immune response. We then investigated whether FOXO3 mediated inhibition of IL-10 and the subsequent activation of M1 macrophages exert a protective effect against mycobacterial infection. We examined the intracellular burden of BCG in macrophages treated with MK and found that such treatment decreased the intracellular loads of BCG by ~60% as compared to the untreated cells (Figure 5D), suggesting that FOXO3 activation boost the macrophage defense mechanisms against invading mycobacteria.

### Silencing of FOXO3 in Macrophages Modulates Th1 Immune Response Against Mycobacterial Infection

**Figure 6:**
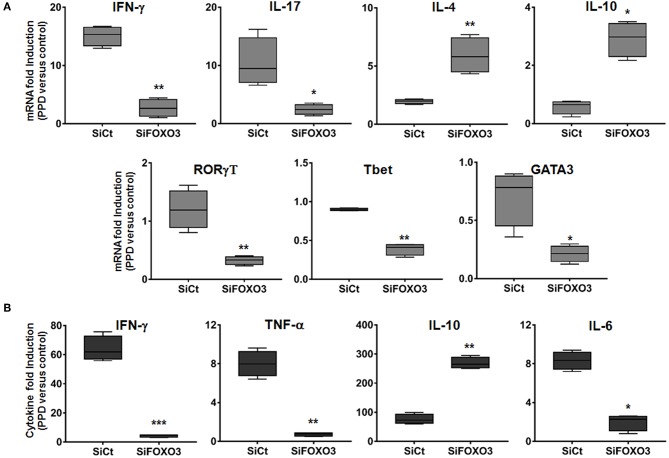
Silencing of FOXO3 in macrophages modulates Th1 immune response against mycobacterial infection. Human monocytes were transfected either with SiCT or SiFOXO3 for 48 h followed by PPD stimulation and incubation with the autologous lymphocytes for 4 days. **(A)** Cells were then collected and tested for the expression levels of the indicated genes by qRT-PCR. **(B)** Supernatants were collected and analyzed by ELISA for IFN-γ, TNF-α, IL-10, and IL-6 secretion. The results are shown in box and whiskers (min to max values) from three independent experiments (i.e., MDMs from 3 different donors), each one carried out in triplicate. Asterisks indicate statistical significance (*p < 0.05, **p < 0.001, ***p < 0.0001).

**Figure 7:**
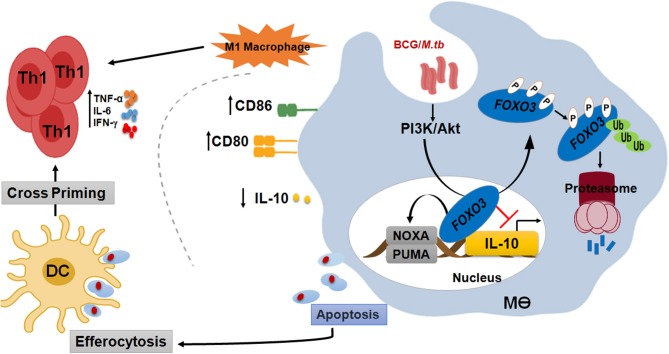
FOXO3, a signaling hub for a protective immune response against mycobacterial infection. In order to compromise host resistance, *M.tb* predominantly activates the pro-survival pathway PI3K/Akt to inhibit apoptosis and induce IL-10 through FOXO3 inhibition. Activated Akt will then phosphorylate the pro-apoptotic transcription factor FOXO3 at three residues, Thr32, Ser253, and Ser315, leading to its translocation to the cytosol where it is stored as a complex FOXO3/chaperone protein 14-3-3 and/or degraded by the proteasome. However, PI3K/Akt inhibition leads to activation of FOXO3, which will accumulate inside the nucleus and restrict IL-10 secretion through direct binding to its proximal promoter. Inhibition of IL-10 signaling is necessary to restore the anti-mycobacterial capacity of the host and increase the polarization of macrophage toward a proinflammatory M1 phenotype, which is crucial for the shaping of an effective Th1 adaptive response against *M.tb* pathogenesis. Activation of FOXO3 would also induce the expression of PUMA and NOXA, the two effectors of macrophage apoptosis. Apoptotic bodies charged with mycobacterial antigens will be ingested by the bystander dendritic cells (DC) to better cross-prime T cells. In conclusion, activated FOXO3 in infected macrophages benefit the protection against TB in many ways. (i) Induces apoptosis of infected-macrophages and disrupts the intracellular replication of *M.tb* within the host. (ii) Inhibits IL-10 and restore the antimicrobial host responses. (iii) Boost the adaptive immune response to *M.tb*.

Activated M1 macrophages are considered potent effector cells, which promote Th1 immune responses while M2 macrophages have immunoregulatory functions and participate in polarized Th2 immune responses (38–40). We then assessed the impact of FOXO3-mediated regulation of macrophage phenotype on the T cells adaptive immune response. As shown in Figure 6, we found that co-culture of PPD-treated and SiFOXO3-transfected human monocytes with their autologous lymphocytes, generated significantly less IFN-γ mRNA in comparison to monocytes that were transfected with the control SiRNA (*p* = 0.0030). Quantification of IFN-γ by ELISA further corroborated this result (Figure 6B, *p* < 0.001). Similarly, the PPD-induced expression of IL-17 gene was significantly reduced when FOXO3 was silenced in monocytes (Figure 6A, *p* = 0.022). In concordance with reduced levels of Th1 cytokines, a clear reduction in PPD-induced gene expression levels of the Th1 related transcription factors RORγT (*p* = 0.0074), Tbet (*p* = 0.004), and GATA3 (*p* = 0.031) was also observed when FOXO3 level was reduced in presenting monocytes. Conversely, this transcription signature was associated with a significant increase in IL-4 (*p* = 0.008) and IL-10 (*p* = 0.020) gene expression levels and a clear reduction in PPD-induced secretion of TNF-α and IL-6 (Figures 6A,B). Overall, these data suggest that FOXO3 inactivation in macrophages enhances IL-10 expression, leading to a modulated M1/Th1 immune response, while active FOXO3 would enhance such immune response against mycobacterial infection, through the inhibition of IL-10 expression in macrophages (Figure 7).

## Discussion

Macrophages represent the forefront of innate immune defense against *M.tb*, which response has major impact on the outcome of infection (41). However, *M.tb* has adopted remarkable strategies to circumvent host defenses. Amongst the many known evasion mechanisms, *M.tb* indeed triggers rapid induction of the anti-inflammatory cytokine IL-10. Several studies have demonstrated tight association of IL-10 secretion upon *M.tb* infection with the recurrence of the disease. Therefore, it is quiet important to understand the molecular mechanisms underlying regulation of IL-10 expression in mycobacteria-infected macrophages. In the present study, we describe the role of FOXO3 transcription factor in the regulation of IL-10 expression in mycobacteria-infected macrophages and the impact of such regulation on adaptive immune response.

Here, we first observed that IL-10 expression is positively regulated by the PI3K/Akt pathway in BCG-infected macrophages. Indeed, inhibition of either PI3K or Akt by Wort or MK, respectively abrogated the BCG-induced IL-10 production. Our findings are in accordance with a previous report showing that the inhibition of *Porphyromonas gingivalis*—mediated activation of the PI3K/Akt pathway results in an abrogation of IL-10 secretion by human monocytes (25). Further, Méndez-samperio et al., have also demonstrated that the strong BCG-induction of IL-10 expression in epithelial cells is dependent on PI3K/Akt activation (42).

FOXO3 is negatively-regulated target of PI3K/Akt pathway and we have previously reported that it is involved in BCG-induced apoptosis of human macrophages (29). Hence, we hypothesized that FOXO3 transcription factor, might act as the suppressor of IL-10 transcription in BCG-infected macrophages. In support of our hypothesis, we found that MK-mediated inhibition of IL-10 secretion was associated with FOXO3 activation at both transcriptional and protein levels. This data correlates with a previous work showing an association between FOXO3 activation and inhibition of IL-10 transcription in monocytes treated with pharmacological inhibition of Akt1/2 (43). In concordance with this, we observed that knocking-down of FOXO3 expression, using specific siRNA, resulted in a significant increase of BCG-induced transcription and secretion of IL-10. This data is in agreement with a previous study that reported a higher expression of IL-10 by neutrophils, monocytes and macrophages from FOXO3^−/−^ mice infected with *Salmonella typhimurium*, as compared to non-infected mice (44). Moreover, it was shown that T cells from FOXO3^−/−^ mice express higher levels of IL-10 compared to wild type ones (45). Our data also correlate with a previous study on dendritic cells where FOXO1, analog of FOXO3, was found to inhibit LPS induced IL-10 production (46). Moreover, FOXO1 deletion in dendritic cells resulted in reduced DC activation and affected the adaptive immune response to bacterial challenge (47, 48). However, these studies did not investigate the mechanisms by which FOXO3 regulates IL-10 expression. We have then performed an *in silico* analysis of IL-10 promoter and identified four forkhead potential binding motifs with a typical FOXO3 binding site at −203 position. We further confirmed the negative regulation of IL-10 by FOXO3 using the reporter gene assay and highlighted the crucial role of the motif at −203 position, which directly binds to FOXO3 protein. Altogether, these data clearly show, for the first time, a negative regulation, by FOXO3, of IL-10 production in mycobacteria-infected macrophages through the direct binding to and interference with IL-10 promoter activity.

During disease progression, *M.tb* induces the re-polarization of macrophages toward the immunomodulatory M2 phenotype (36) through induction of high level of IL-10, leading to a Th2 immune response, which correlates with the severity of the disease (37). In order to assess the impact of FOXO3-mediated inhibition of IL-10 on macrophage polarization, the expression levels of the two typical M1 surface markers CD80 and CD86 was assessed, during progress of the adaptive immune response against mycobacterial antigens. As expected, we found a significant decrease of the expression levels of the two studied markers in PPD-treated and SiFOXO3-transfected macrophages, compared to the Si-control-transfected ones. Conversely, we observed that pharmacological activation of FOXO3, in BCG-infected and non-infected macrophages, resulted in the up-regulation of CD86 expression along with MHC class II and class I, two additional markers of M1 macrophages. These observations suggest that, by inhibiting the IL-10 expression in mycobacteria-infected macrophages, FOXO3 ameliorates the presenting activity and favors the polarization of host macrophages toward the pro-inflammatory M1 phenotype during TB immune response. In concordance, we observed that FOXO3 silencing in monocytes results in the modulation of Th1 related markers and the induction of a Th2 response, against mycobacterial antigens. The only discrepancy we found was the decrease in GATA3 expression associated to the upregulation of IL-4 after FOXO3 silencing in macrophages. This could be explained by the potential regulation of GATA3 transcription by FOXO3. Indeed, a previous study has proposed the positive regulation of GATA3 by FOXO1 (49), an isoform of FOXO3 with which it shares common targets (50, 51).

Taken together, our data suggest that FOXO3 acts as an inducer of a protective M1/Th1 immune response through the negative regulation of IL-10 expression in infected macrophages. Our data correlate with the previous reported protective role of FOXO3 against *Salmonella typhimurium (ST)* infection. Indeed, FOXO3 signaling inhibited IL-10 secretion with a concomitant induction of inflammatory immune responses in mice infected with ST, leading to the control of the intracellular bacteria (44). Several studies pointed that *M.tb* successfully engages activators of IL-10 transcription such as STAT3 and ReIB during infection to abolish the protective response mounted by the host and ensures its survival and persistence (11, 52). Our data suggest that *M.tb* might inactivate FOXO3 as an additional mechanism to enhance IL-10 secretion in order to establish persistent infection in the host. This is in agreement with previous studies that reported an abrogation of FOXO3 expression in active TB patients, compared to healthy controls (30, 53). It has been also shown that an SNP affecting FOXO3 expression was associated with active TB in a dominant inheritance mode (54). Taken together, our study presents a strong evidence that FOXO3 constitutes a major signaling hub that is manipulated by mycobacterial species and that disruption of FOXO3 signaling might affect the regulation of anti-*M.tb* directed immune response.

In order to compromise host resistance, *M.tb* predominantly activates the pro-survival pathway PI3K/Akt to inhibit apoptosis and induce IL-10 through FOXO3 inhibition. Activated Akt will then phosphorylate the pro-apoptotic transcription factor FOXO3 at three residues, Thr32, Ser253, and Ser315, leading to its translocation to the cytosol where it is stored as a complex FOXO3/chaperone protein 14-3-3 and/or degraded by the proteasome. However, PI3K/Akt inhibition leads to activation of FOXO3, which will translocate to the nucleus and restrict

IL-10 secretion through direct binding to its proximal promoter. Inhibition of IL-10 signaling is necessary to restore the anti-mycobacterial capacity of the host and increases the polarization of macrophage toward a proinflammatory M1 phenotype, which is crucial for the shaping of an effective Th1 adaptive response against *M.tb* pathogenesis. Activation of FOXO3 would also induce macrophage apoptosis. Apoptotic bodies charged with mycobacterial antigens will be then ingested by the bystander dendritic cells (DC) to better cross-prime T cells (Figure 7).

The dual functions of FOXO3 as an inducer of macrophage apoptosis and inhibitor of IL-10 secretion, suggest that this factor can be proposed as a new target for host-directed therapy against Tuberculosis. Several commercially available Akt inhibitors, like the used MK2206, act through FOXO3 activation are in pre- and clinical use to treat cancers (55, 56). Based on the results obtained, we suggest that these FOXO3 activators may be used as adjunct to shorten the duration of therapy against drug-resistant TB. This may lead to enhanced repression of IL-10 secretion and higher apoptosis, thereby generating a stronger immune response and a more rapid clearance of *M.tb* infection. This hypothesis is in agreement with a previous study where authors reported that AKT1 inhibitors prevented intracellular growth of various bacteria including multi drug resistant strains of *M.tb* (57). In the other hand, targeting FOXO3 would be also useful to augment the efficacy of BCG. Indeed, activation of FOXO3 during vaccination would inhibit the BCG-induced secretion of IL-10 and enhance apoptosis of infected macrophages, leading to a stronger Th1 protective immune response against *M.tb* (Figure 7).

Overall, we have identified FOXO3 as a transcription factor that tunes the host inflammatory response during mycobacterial infection through the regulation of IL-10 expression. These findings advance our understanding of protective immunity to *M.tb* by revealing the importance of FOXO3 transcription factor, which deserves further investigation as it may be used as therapeutic/preventive target against TB.

## Data Availability Statement

All datasets generated for this study are included in the article/Supplementary Material.

## Ethics Statement

The studies involving human participants were reviewed and approved by the institutional ethics committee of Institut Pasteur de Tunis. The Healthy Volunteers provided their written informed consent to participate in this study. The animal study was reviewed and approved by the Institutional animal ethics committee of THSTI, according to the control and supervision on animals (CPCSEA) guideline.

## Author Contributions

ME, RB, and MH conceived and designed the experiments. ME, RB, MH, and RS performed and analyzed the data, interpreted them, and wrote the paper as well. M-RB contributed with providing reagents and discussing the manuscript.

### Conflict of Interest

The authors declare that the research was conducted in the absence of any commercial or financial relationships that could be construed as a potential conflict of interest.

## References

[1] Macneil A Glaziou P Sismanidis C Maloney S Floyd K. Global epidemiology of tuberculosis and progress toward achieving global targets — 2017. MMWR Morb Mortal Wkly Rep. (2019) 68:263–6. 10.15585/mmwr.mm6811a3 30897077 PMC6478060

[2] Pitt JM Blankley S McShane H O'Garra A. Vaccination against tuberculosis: how can we better BCG? Microb Pathog. (2013) 58:2–16. 10.1016/j.micpath.2012.12.002 23257069

[3] Wallis RS Hafner R. Advancing host-directed therapy for tuberculosis. Nat Rev Immunol. (2015) 15:255–63. 10.1038/nri3813 25765201

[4] Li G Liu G Song N Kong C Huang Q Su H. A novel recombinant BCG-expressing pro-apoptotic protein BAX enhances Th1 protective immune responses in mice. Mol Immunol. (2015) 66:346–56. 10.1016/j.molimm.2015.04.003 25942359

[5] Sia JK Georgieva M Rengarajan J. Innate immune defenses in human tuberculosis: an overview of the interactions between *Mycobacterium tuberculosis* and innate immune cells. J Immunol Res. (2015) 2015:747543. 10.1155/2015/747543 26258152 PMC4516846

[6] van Crevel R Ottenhoff THM van der Meer JWM. Innate immunity to *Mycobacterium tuberculosis*. Clin Microbiol Rev. (2002) 15:294–309. 10.1128/CMR.15.2.294-309.2002 11932234 PMC118070

[7] Domingo-gonzalez R Prince O Cooper A Khader SA. Cytokines and chemokines in *Mycobacterium tuberculosis* infection. In: Tuberculosis and the Tubercle Bacillus. 2nd ed. Washington, DC: ASM Press (2016). p. 33–72. 10.1128/microbiolspec.TBTB2-0018-2016 PMC5205539 27763255

[8] Verbon A Juffermans N Van deventer SJH Speelman P Van Deutekom H. Serum concentrations of cytokines in patients with active tuberculosis (TB) and after treatment. Clin Exp Immunol. (1999) 115:110–3. 10.1046/j.1365-2249.1999.00783.x 9933428 PMC1905191

[9] Boussiotis VA Tsai EY Yunis EJ Thim S Delgado JC Dascher CC. IL-10 – producing T cells suppress immune responses in anergic tuberculosis patients. J Clin Invest. (2000) 105:1317–24. 10.1172/JCI9918 10792007 PMC315449

[10] Redpath S Ghazal P Gascoigne NRJ. Hijacking and exploitation of IL-10 by intracellular pathogens. Trends Microbiol. (2001) 9:86–92. 10.1016/S0966-842X(00)01919-3 11173248

[11] O'Leary S O'Sullivan MP Keane J. IL-10 blocks phagosome maturation in *Mycobacterium tuberculosis*-infected human macrophages. Am J Resp Cell Mol Biol. (2011) 45:172–80. 10.1165/rcmb.2010-0319OC 20889800

[12] Faulkner L Buchan G Baird M Interleukin-10 does not affect phagocytosis of particulate antigen by bone marrow-derived dendritic cells but does impair antigen presentation. Immunology. (2000) 99:523–31. 10.1046/j.1365-2567.2000.00018.x 10792499 PMC2327185

[13] Bakhru P Sirisaengtaksin N Soudani E Mukherjee S Khan A Jagannath C. BCG vaccine mediated reduction in the MHC-II expression of macrophages and dendritic cells is reversed by activation of Toll-like receptors 7 and 9. Cell Immunol. (2014) 287:53–61. 10.1016/j.cellimm.2013.11.007 24384074 PMC4096037

[14] Antoniv TT Ivashkiv LB. Interleukin-10-induced gene expression and suppressive function are selectively modulated by the PI3K-Akt-GSK3 pathway. Immunology. (2011) 132:567–77. 10.1111/j.1365-2567.2010.03402.x 21255011 PMC3075510

[15] Abdul-Aziz M Tsolaki AG Kouser L Carroll MV Al-Ahdal MN Sim RB. Complement factor H interferes with *Mycobacterium bovis* BCG entry into macrophages and modulates the pro-inflammatory cytokine response. Immunobiology. (2016) 221:944–52. 10.1016/j.imbio.2016.05.011 27262511

[16] Lopes RL Borges TJ Zanin RF Bonorino C. IL-10 is required for polarization of macrophages to M2-like phenotype by mycobacterial DnaK (heat shock protein 70). Cytokine. (2016) 85:123–9. 10.1016/j.cyto.2016.06.018 27337694

[17] Cyktor JC Carruthers B Kominsky RA Beamer GL Stromberg P Turner J. IL-10 inhibits mature fibrotic granuloma formation during *Mycobacterium tuberculosis* infection. J Immunol. (2013) 190:2778–90. 10.4049/jimmunol.1202722 23396944 PMC3594073

[18] Beamer GL Flaherty DK Assogba BD Stromberg P Gonzalez-Juarrero M de Waal Malefyt R. Interleukin-10 promotes *Mycobacterium tuberculosis* disease progression in CBA/J mice. J Immunol. (2008) 181:5545–50. 10.4049/jimmunol.181.8.5545 18832712 PMC2728584

[19] Redford PS Boonstra A Read S Pitt J Graham C Stavropoulos E. Enhanced protection to *Mycobacterium tuberculosis* infection in IL-10-deficient mice is accompanied by early and enhanced Th1 responses in the lung. Eur J Immunol. (2010) 40:2200–10. 10.1002/eji.201040433 20518032 PMC3378704

[20] Redford PS Murray PJ O'Garra A. The role of IL-10 in immune regulation during *M. tuberculosis* infection. Mucosal Immunol. (2011) 4:261–70. 10.1038/mi.2011.7 21451501

[21] Jamil B Shahid F Hasan Z Nasir N Razzaki T Dawood G Interferonγ/IL10 ratio defines the disease severity in pulmonary and extra pulmonary tuberculosis. Tuberculosis. (2007) 87:279–87. 10.1016/j.tube.2007.03.004 17532265

[22] Skolimowska KH Rangaka MX Meintjes G Pepper DJ Seldon R Matthews K. Altered ratio of IFN-γ/IL-10 in patients with drug resistant *Mycobacterium tuberculosis* and HIV- tuberculosis immune reconstitution inflammatory syndrome. PLoS ONE. (2012) 7:e46481. 10.1371/journal.pone.0046481 23071578 PMC3468619

[23] Pitt JM Stavropoulos E Redford PS Beebe AM Bancroft GJ Young DB. Blockade of IL-10 signaling during bacillus calmette-guerin vaccination enhances and sustains Th1, Th17, and innate lymphoid IFN- and IL-17 responses and increases protection to *Mycobacterium tuberculosis* infection. J Immunol. (2012) 189:4079–87. 10.4049/jimmunol.1201061 22972927 PMC3467194

[24] Dallenga T. Strategies to improve vaccine efficacy against Tuberculosis by Targeting innate immunity. Front Immunol. (2017) 8:1755. 10.3389/fimmu.2017.01755 29312298 PMC5732265

[25] Martin M Schifferle RE Cuesta N Vogel SN Katz J Michalek SM. Role of the phosphatidylinositol 3 kinase-Akt pathway in the regulation of IL-10 and IL-12 by Porphyromonas gingivalis lipopolysaccharide. J Immunol. (2003) 171:717–25. 10.4049/jimmunol.171.2.717 12847238

[26] Bai W Liu H Ji Q Zhou Y Liang L Zheng R. TLR3 regulates mycobacterial RNA-induced IL-10 production through the PI3K/AKT signaling pathway. Cell Signal. (2014) 26:942–50. 10.1016/j.cellsig.2014.01.015 24462705

[27] Stanley SA Barczak AK Silvis MR Luo SS Sogi K Vokes M. Identification of host-targeted small molecules that restrict intracellular *Mycobacterium tuberculosis* growth. PLoS Pathog. (2014) 10:e1003946. 10.1371/journal.ppat.1003946 24586159 PMC3930586

[28] Hwang J-W Rajendrasozhan S Yao H Chung S Sundar IK Huyck HL. FOXO3 deficiency leads to increased susceptibility to cigarette smoke-induced inflammation, airspace enlargement, and chronic obstructive pulmonary disease. J Immunol. (2011) 187:987–98. 10.4049/jimmunol.1001861 21690325 PMC3131437

[29] Haoues M Refai A Mallavialle A Barbouche MR Laabidi N Deckert M. Forkhead box O3 (FOXO3) transcription factor mediates apoptosis in BCG-infected macrophages. Cell Microbiol. (2014) 16:1378–90. 10.1111/cmi.12298 24712562

[30] Huang J Jiao J Xu W Zhao H Zhang C Shi Y. MiR-155 is upregulated in patients with active tuberculosis and inhibits apoptosis of monocytes by targeting FOXO3. Mol Med Rep. (2015) 12:7102–8. 10.3892/mmr.2015.4250 26324048

[31] Salkind N Encyclopedia of Measurement and Statistics. Thousand Oaks, CA: SAGE Publishing (2007). 10.4135/9781412952644

[32] Morgan CJ. Use of proper statistical techniques for research studies with small samples. Am J Physiol. (2017) 313:L873–7. 10.1152/ajplung.00238.2017 28982734

[33] Stroeken P Versteeg R Koster J Santo EE Sluis PV Westerhout EM. FOXO3a is a major target of inactivation by PI3K/AKT signaling in aggressive neuroblastoma. Cancer Res. (2013) 73:2189–98. 10.1158/0008-5472.CAN-12-3767 23378341

[34] Iida M Brand TM Campbell DA Starr MM Luthar N Traynor AM. Targeting AKT with the allosteric AKT inhibitor MK-2206 in non-small cell lung cancer cells with acquired resistance to cetuximab. Cancer Biol Therapy. (2013) 14:481–91. 10.4161/cbt.24342 23760490 PMC3813564

[35] Day J Friedman A Schlesinger LS. Modeling the immune rheostat of macrophages in the lung in response to infection. Proc Natl Acad Sci USA. (2009) 106:11246–51. 10.1073/pnas.0904846106 19549875 PMC2708732

[36] Refai A Gritli S Barbouche M Essafi M. *Mycobacterium tuberculosis* virulent factor ESAT-6 drives macrophage differentiation toward the pro-inflammatory M1 phenotype and subsequently switches it to the anti-inflammatory M2 phenotype. (2018) 8:1–14. 10.3389/fcimb.2018.00327 30283745 PMC6157333

[37] Lugo-Villarino G Vérollet C Maridonneau-Parini I Neyrolles O. Macrophage polarization: convergence point targeted by *Mycobacterium tuberculosis* and HIV. Front Immunol. (2011) 2:43. 10.3389/fimmu.2011.00043 22566833 PMC3342390

[38] Murray PJ. Macrophage polarization. Annu Rev Physiol. (2017) 79:541–66. 10.1146/annurev-physiol-022516-034339 27813830

[39] Martinez FO Gordon S. The M1 and M2 paradigm of macrophage activation: time for reassessment. F1000prime Rep. (2014) 6:13. 10.12703/P6-13 24669294 PMC3944738

[40] Sica A Erreni M Allavena P Porta C. Macrophage polarization in pathology. Cell Mol Life Sci. (2015) 72:4111–26. 10.1007/s00018-015-1995-y 26210152 PMC11113543

[41] Queval CJ Brosch R Simeone R. The macrophage: a disputed fortress in the battle against *Mycobacterium tuberculosis*. Front Microbiol. (2017) 8:2284. 10.3389/fmicb.2017.02284 29218036 PMC5703847

[42] Méndez-samperio P Trejo A Pérez A. *Mycobacterium bovis* bacillus calmette – Guérin (BCG) stimulates IL-10 production via the PI3K/Akt and p38 MAPK pathways in human lung epithelial cells. Cell Immunol. (2008) 251:37–42. 10.1016/j.cellimm.2008.03.002 18423589

[43] Over B Ziegler S Foermer S Weber ANR Bode KA Heeg K. IRAK4 turns IL-10+ phospho-FOXO+ monocytes into pro-inflammatory cells by suppression of protein kinase B. Eur J Immunol. (2013) 43:1630–42. 10.1002/eji.201243217 23519847

[44] Joseph J Ametepe ES Haribabu N Agbayani G Krishnan L Blais A. Inhibition of ROS and upregulation of inflammatory cytokines by FoxO3a promotes survival against *Salmonella typhimurium*. Nat Commun. (2016) 7:12748. 10.1038/ncomms12748 27599659 PMC5023958

[45] Lin L Hron JD Peng SL. Regulation of NF-κB, Th activation, and autoinflammation by the forkhead transcription factor Foxo3a. Immunity. (2004) 21:203–13. 10.1016/j.immuni.2004.06.016 15308101

[46] Chung S Ranjan R Lee YG Park GY Karpurapu M Deng J. Distinct role of FoxO1 in M-CSF- and GM-CSF-differentiated macrophages contributes LPS-mediated IL-10 : implication in hyperglycemia. J Leukoc Biol. (2015) 97:327–39. 10.1189/jlb.3A0514-251R 25420919 PMC4304426

[47] Dong G Wang Y Xiao W Pacios S Xu F Tian C. FOXO1 regulates dendritic cell activity through ICAM-1 and CCR7. J Immunol. (2015) 194:3745–55. 10.4049/jimmunol.1401754 25786691 PMC4390509

[48] Xiao W Dong G Pacios S Alnammary M Barger LA Wang Y. FOXO1 deletion reduces dendritic cell function and enhances susceptibility to periodontitis. Am J Pathol. (2015) 185:1085–93. 10.1016/j.ajpath.2014.12.006 25794707 PMC4380851

[49] Wu YY Elshimali Y Sarkissyan M Mohamed H Clayton S Vadgama J Abstract 704: expression of FOXO1 is associated with GATA3 and Annexin-1 and predicts disease-free survival in breast cancer. Cancer Res. (2012) 2:704 10.1158/1538-7445.AM2012-704 PMC3236574 22206049

[50] Greer EL Brunet A. FOXO transcription factors at the interface between longevity and tumor suppression. Oncogene. (2005) 24:7410–25. 10.1038/sj.onc.1209086 16288288

[51] Dejean AS Hedrick SM Kerdiles YM. Highly specialized role of forkhead Box O transcription factors in the immune system. Antioxid Redox Signal. (2011) 14:663–74. 10.1089/ars.2010.3414 20673126 PMC3021368

[52] Queval CJ Song OR Deboosère N Delorme V Debrie AS Iantomasi R. STAT3 represses nitric oxide synthesis in human macrophages upon *Mycobacterium tuberculosis* infection. Sci Rep. (2016) 6:29297. 10.1038/srep29297 27384401 PMC4935992

[53] Xi X Zhang C Han W Zhao H Zhang H Jiao J. MicroRNA-223 is upregulated in active tuberculosis patients and inhibits apoptosis of macrophages by targeting FOXO3. Genet Test Mol Biomark. (2015) 19:650–6. 10.1089/gtmb.2015.0090 26505221

[54] Lu Y Zhu Y Wang X Wang F Peng J Hou H. FOXO3 rs12212067: T > G association with active tuberculosis in han Chinese population. Inflammation. (2016) 39:10–5. 10.1007/s10753-015-0217-y 26223437

[55] Yang J Hung M Molecular pathways a new fork for clinical application : T argeting forkhead T ranscription factors in cancer. Clin Cancer Res. (2009) 15:752–8. 10.1158/1078-0432.CCR-08-0124 19188143 PMC2676228

[56] Chandarlapaty S Sawai A Scaltriti M Rodrik-Outmezguine V Grbovic-Huezo O Serra V. AKT inhibition relieves feedback suppression of receptor tyrosine kinase expression and activity. Cancer Cell. (2011) 19:58–71. 10.1016/j.ccr.2010.10.031 21215704 PMC3025058

[57] Kuijl C Savage NDL Marsman M Tuin AW Janssen L Egan DA. Intracellular bacterial growth is controlled by a kinase network around PKB/AKT1. Nature. (2007) 450:725–30. 10.1038/nature06345 18046412

