# Supplementary material for: FOXO3 Transcription Factor Regulates IL-10 Expression in Mycobacteria-Infected Macrophages, Tuning Their Polarization and the Subsequent Adaptive Immune Response
Source: Front Immunol. 2019 Dec 12;10:2922. doi: 10.3389/fimmu.2019.02922 (PMC6927284; doi:10.3389/fimmu.2019.02922)
Supplement: Supplementary file 2 [file Data_Sheet_1.docx]

**Table S1:** Sequences of the used primers to quantify human gene expression by RT-qPCR.

| Gene | **Primer** | **Sequence (5'-3')** |
| --- | --- | --- |
| GAPDH | Forward  Reverse | GGATTTGGTCGTATTGGG  GGAAGATGGTGATGGGATT |
| *β-*ACTIN: | Forward  Reverse | CACCAACTGGGACGACAT  ACAGCCTGGATAGCAACG |
| FOXO3 | Forward  Reverse | AAATGAAAGCTCACTCTGGATTCC TGTGCAATTCCTATG CAATC |
| IL-10 | Forward  Reverse | TCAAGGCGCATGTGAACTCC  GATGTCAAACTCACTCATGT |
| IFN-γ | Forward  Reverse | TTTGGGTTCTCTTGGCTGTT  TCCATTATCCGCTACATCTGAA |
| IL-17a | Forward  Reverse | ACCAATCCCAAAAGGTCCTC  TGGATGGGGACAGAGTTCAT |
| CD80 | Forward  Reverse | CAGGGAACATCACCATCCAA CAGCGTTGCCACTTCTTTCA |
| CD86 | Forward  Reverse | AGCGGCCTCGCAACTCTTAT AAAACACGCTGGGCTTCATC |
| IL-4 | Forward  Reverse | CACGGACACAAGTGCGATA  GATGTCTGTTACGGTCAACTCG |
| GATA3 | Forward  Reverse | CAAAATGAACGGACAGAACCG  GCTCTCCTGGCTGCAGACA |
| RORγT | Forward  Reverse | AATGACCAGATTGTGCTTCTCAAAG  GGTTGTCAGCATTGTAGGCCC |
| Tbet | Forward  Reverse | AGGATTCCGGGAGAACTTTGAG  AATTGACAGTTGGGTCCAGGC |

**Table S2:** Sequences of the used primers to quantify murine gene expression by RT-qPCR.

| Gene | **Primer** | **Sequence (5'-3')** |
| --- | --- | --- |
| GAPDH | Forward  Reverse | ACCACAGTCCATGCCATCAC  CACCACCCTGTTGCTGTAGCC |
| FOXO3 | Forward  Reverse | CTGGGGGAACCTGTCCTATG  TCATTCTGAACGCGCATGAAG |
| IL-10 | Forward  Reverse | \| GGTTGCCAAGCCTTATCGGA \| \| --- \| \| GGGGAGAAATCGATGACAGC \| |
| Arg-1 | Forward  Reverse | ATGGAAGAGACCTTCAGCTAC  GCTGTCTTCCCAAGAGTTGGG |

**Table S3:** Sequences of the used primers to disrupt FOXO3 binding motifs on the human IL-10 promoter construct.

| Position on IL-10 promoter | Primer sequence (5'-3') |
| --- | --- |
| -526 | **F** GCCCACCCCCTCATT**T**CTTGGGGAAACTAAG  **R** CTTAGTTTCCCCAAG**A**AATGAGGGGGTGGGC |
| -303 | **F** CTGTTCTCCCCAGGA**C**AACTTTTTTTAATTGAG  **R** CTCAATTAAAAAAAGTT**G**TCCTGGGGAGAACAG |
| -203 | **F** GTTCAACCAATCATTTT**T**TACGATGCAAAAATTG  **R** CAATTTTTGCATCGTA**A**AAAATGATTGGTTGAAC |
| -17 | **F** CTCTTGCAAAACCAAAC**A**AGACAGACTTGCAAAA  **R**  CTCTTGCAAAACCAAAC**A**AGACAGACTTGCAAAA |
