# Supplementary figures and images for: FOXO3 Transcription Factor Regulates IL-10 Expression in Mycobacteria-Infected Macrophages, Tuning Their Polarization and the Subsequent Adaptive Immune Response
Source: Front Immunol. 2019 Dec 12;10:2922. doi: 10.3389/fimmu.2019.02922 (PMC6927284; doi:10.3389/fimmu.2019.02922)

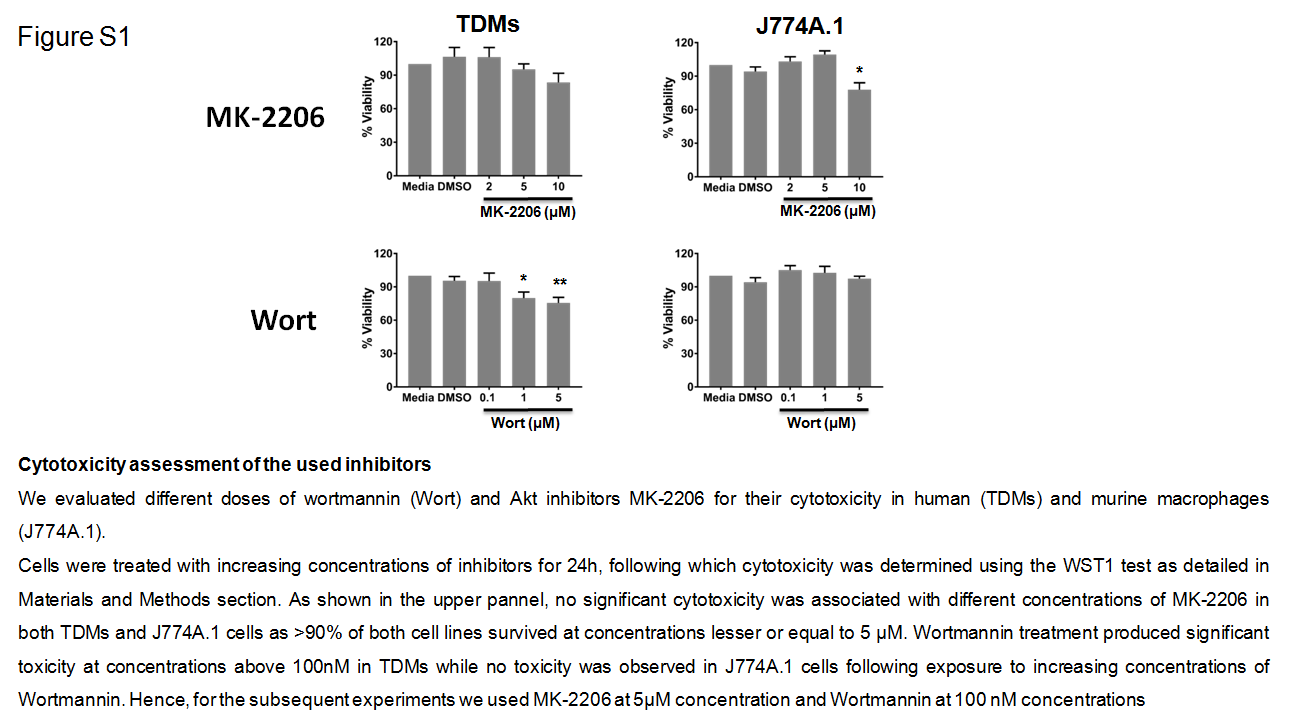

Supplement: Supplementary file 3 [file Image_1.TIF]

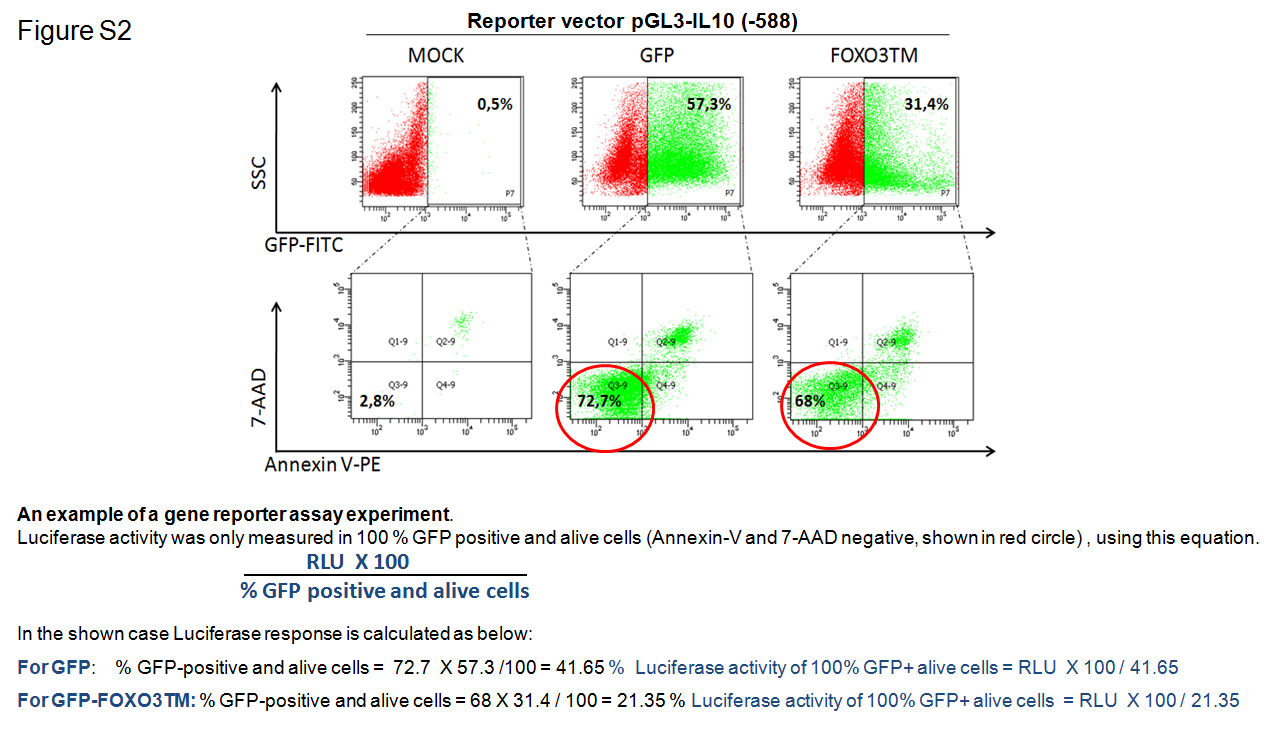

Supplement: Supplementary file 4 [file Image_2.TIF]

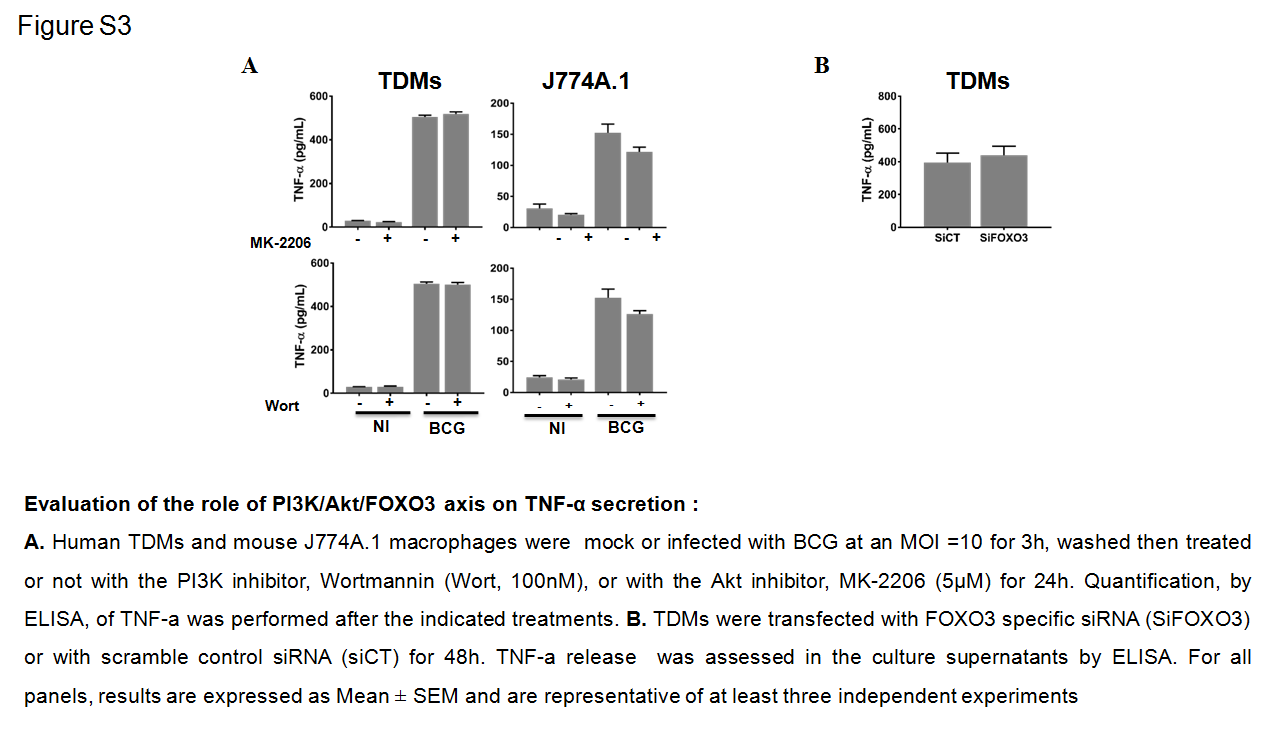

Supplement: Supplementary file 5 [file Image_3.TIF]

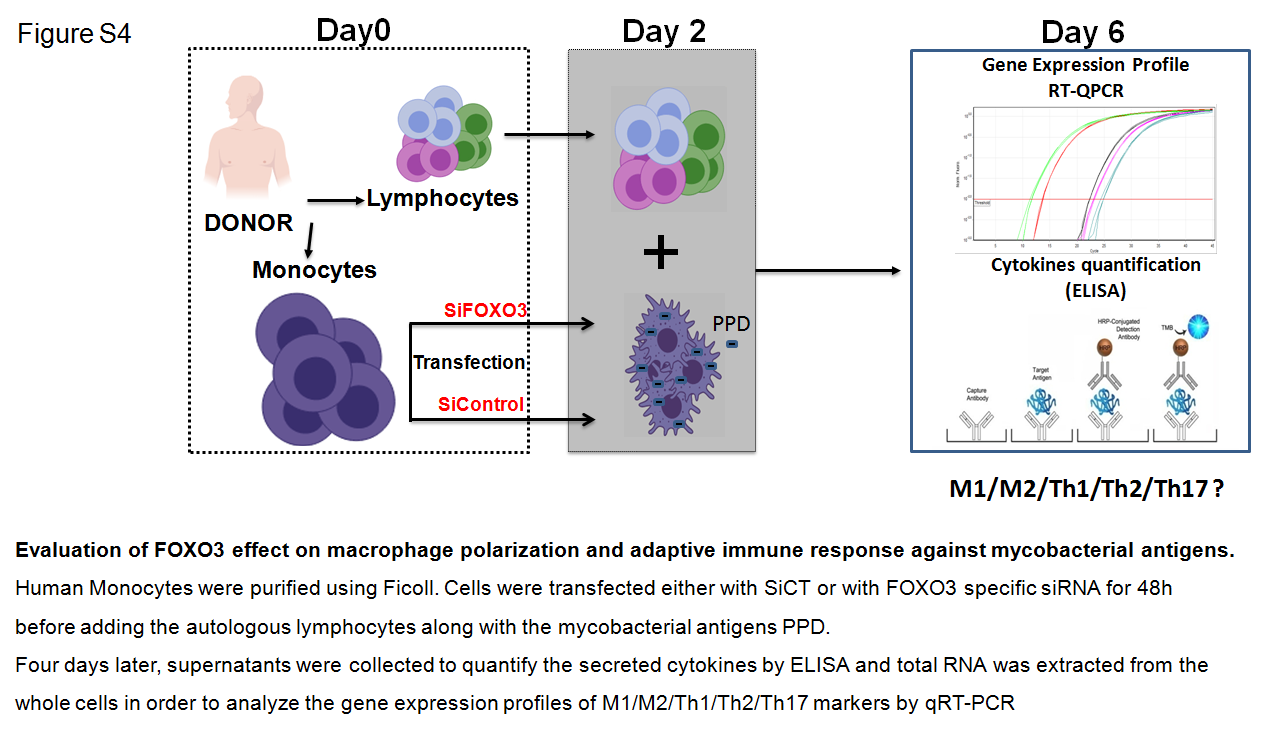

Supplement: Supplementary file 6 [file Image_4.TIF]
